# Paving the way to improve therapy for Myeloproliferative Neoplasms

**DOI:** 10.1038/s41467-022-32694-2

**Published:** 2022-08-26

**Authors:** Megan Bywater, Steven W. Lane

**Affiliations:** 1 Cancer Program, QIMR Berghofer MRI, Brisbane, QLD 4006 Australia; 2 School of Medicine, University of Queensland, Brisbane, QLD 4006 Australia; 3 Cancer Care Services, Royal Brisbane and Women’s Hospital, Brisbane, QLD 4029 Australia

**Keywords:** Experimental models of disease, Drug development, Mechanisms of disease, Haematological cancer

## Abstract

Long-acting IFNα induces durable molecular responses in myeloproliferative neoplasms. Emerging studies, including Saleiro et al. recently published in *Nature Communications*, have identified promising candidates that may synergise with IFNα by targeting stem cell function or feedback loops that mediate treatment resistance.

## Clinical management in Myeloproliferative Neoplasms

The MyeloProliferative Neoplasms (MPN) are characterised by the excess production of phenotypically normal mature myeloid cells or cell products, specifically red blood cells in polycythemia vera (PV), megakaryocytes and platelets in essential thrombocythemia (ET) and additional cytokine driven fibro-cellular infiltration of the bone marrow in primary myelofibrosis (PMF)^1^. This expansion of mature myeloid cell populations is driven by the constitutive activation of the JAK-STAT signalling pathway in committed myeloid progenitors as a consequence of mutations in either *JAK2*, *MPL* or *CALR*^1^. However, although this disease is phenotypically driven from expanded committed myeloid progenitor cell compartments, these populations remain untransformed as they lack the capacity for long-term self-renewal. As a consequence, MPN-driving mutations must be maintained within the haematopoietic stem cell (HSC) compartment^2^.

MPNs are long-term chronic conditions and patient management is focussed on ameliorating the symptoms related to clinical pathologies^1^. Current approaches are limited by toxicity of long-term treatments that have little disease modifying activity and do not prevent transformation to more aggressive diseases such as leukaemia. Venesection leads to iron restricted erythropoiesis and is intended to reduce the expanded mature red blood cell population. To a similar end, hydroxycarbamide (also known as hydroxyurea) is frequently used for cytoreduction to control erythrocytosis and thrombocytosis with a concomitant effect on reducing thrombotic tendency. More recently, small molecule inhibitors of JAKs have been developed to target the signalling pathway hyperactivated in this disease. Unfortunately, current studies indicate that the MPN stem cell pool is not reliant on the constitutive activation of JAK2 for survival^3^. Consequently, JAK1/2 inhibitors like Ruxolitinib have proven effective at reducing the excess production of mature myeloid cells, inflammatory cytokine levels and the associated clinical symptoms in MPN, but have had limited efficacy in reducing the size of the MPN stem cell pool^3^. Therefore, long-term disease management via targeted JAK2 inhibition will most likely require chronic administration of these compounds, the practicality of which can be limited by significant side effects.

Disease progression to secondary myelofibrosis (sMF) or acute myeloid leukaemia (sAML) occurs in ~8–20% and 8–26% of patients with ET and PV respectively over a 20-year period^1^ and is related to an expanded mutational spectrum^4^. Importantly, clinical outcomes subsequent to disease progression are poor on account of limited effective treatment options. In the case of sMF and MPN-driver-positive sAML, it is assumed that transformation is driven by the selective pressure provided by the MPN-driving oncogene. Considering this, it would seem the most rational approach for the clinical management of MPN is the development of treatment options that selectively target the MPN stem cell pool to decrease both the burden of the chronic management of this disease and prevent the deleterious outcomes related to disease progression.

## Long-acting IFNα induces durable responses in MPN

Recent years have seen a renaissance in the use of interferon alpha (IFNα) for the treatment of MPN, specifically PV and ET. Initially, the use of IFNα was rationalised on the basis of its known myelosuppressive effects with several groups also postulating an immunostimulatory role of these agents. The clinical uptake of IFNα therapy was initially hampered by low compliance related to a poor pharmacokinetic profile of unmodified recombinant forms. More recently, pegylated versions of IFNα have been found to be more persistent in vivo, extending the duration of response and allowing a longer interval between doses. Several studies have now compared long-acting pegylated IFNα with hydroxycarbamide in inducing durable long-term haematological responses^5–7^, including the normalisation of red blood cell counts and the prevention of thromboembolic events, with the advantage of also being non-leukemogenic. Importantly, IFNα therapy has also proven effective at targeting the MPN stem cell pool with durable molecular remissions also being observed across multiple studies^5–7^.

Mechanistically, IFNα drives cell cycle entry in HSCs with this mitogenic effect being more potent in *Jak2*-mutant HSCs^3,8^ supporting a prevailing hypothesis that molecular remissions observed in PV patients receiving IFNα are due to the preferential functional decline of the MPN stem cell pool. Despite its appreciable clinical success, it is clear that the selectivity of IFNα for mutant MPN stem cells over normal stem cells is mild. Consequently, there is an active field of interest looking to understand this selectivity and exploit it through combination therapies. Recently published in *Nature Communications*, Saleiro et al.^9^ further elucidates the ability of IFNα to activate a PKCd-ULK1-p38 MAPK signalling cascade that acts in parallel to STAT1 to drive transcription of interferon response genes (IRGs) and that genetic disruption of this pathway can attenuate the ability of IFNα to reduce self-renewal in malignant erythroid precursors. In demonstrating that ULK1 preferentially associates with the activated forms of ROCK1/2 and that IFNα also drives ROCK1/2 activation, they postulate whether modulating ROCK1/2 activity directly may also affect cellular responses to IFNα. In support of this, they demonstrate that both genetic and pharmacological inhibition of ROCK1/2 can enhance the ability of IFNα to reduce cell viability in *JAK2*-mutant cell lines and self-renewal in PV patient erythroid precursors^9^. It will be important to determine whether this combination will have enhanced selectivity in the targeting of MPN stem cells over normal stem cells.

Another intriguing combinatorial approach to enhance MPN stem cell selectivity exploits the higher basal levels of PML-nuclear bodies (NB) present in *JAK2V617F* HSCs^10^. Arsenic trioxide (ATO) is the standard of care in acute promyelocytic leukaemia and acts in part through degradation of the driving oncogene PML/RAR alpha. ATO can also drive PML-NB formation, which has been shown to be tumour suppressive. Notably, PML is also an IRG, with the combination of IFNα and ATO proving highly effective in preferentially increasing PML-NB formation in *Jak2*-mutant stem cells and reducing their capacity to transplant disease^10^. Perhaps counterintuitive is the combination of IFNα with the JAK1/2 inhibitor Ruxolitinib, which clearly has clinical activity, although trials have been limited by toxicity^11^. Although both agents have potent activity against *JAK2V617F* MPN, JAK1 kinase activity is required for IFNα-mediated phosphorylation and activation of STAT1. Interestingly, despite being able to robustly inhibit STAT1 phosphorylation in LT-HSCs in response to IFNα in vitro, Ruxolitinib effects only minimal attenuation of STAT1 phosphorylation and cell cycle entry of LT-HSCs in response to IFNα in vivo^3^. These data suggest this combination may be able to exploit the MPN stem cell-selective effects of IFNα in addition to the anti-proliferative and anti-inflammatory effects of Ruxolitinib on MPN myeloid precursors and committed cells.

## Evolving resistance to IFNα mediated by alternate signalling pathways

Saleiro et al.^9^ also postulate the utility of identifying biomarkers of IFNα treatment response in the clinical management of MPN. They show that the hyperactivation of the PKCd-ULK1-p38 MAPK pathway may enhance the therapeutic response to IFNα treatment in MPN, in that increased expression of ULK1 and p38 MAPK mRNA correlates with haematological responses in a combined cohort of IFNα-treated PV and ET patients. Consistent with this, a number of genetic determinants of IFNα treatment response are emerging. Notably, despite the convergent mechanistic pathways of MPN-driver mutations, it appears that the ability of IFNα to deplete the MPN stem cell clone is largely restricted to patients with *JAK2V617F* mutations, and that *CALR* mutant MPN is less likely to achieve molecular remissions in response to pegylated IFNα therapy, despite achieving similar outcomes in terms of haematological responses^12,13^. Early studies also indicate that *DNMT3A* mutations are enriched after IFNα treatment, suggesting a possible association of this mutation with IFNα resistance in patients^14^. As such, we must consider how molecular responses to MPN therapies may be modified by the growing list of concomitant mutations in this disease^4,15^. To achieve this it is imperative that recent large cohort studies comparing the efficacy of IFNα therapy to standard of care should be combined with Next Generation Sequencing analysis of pathogenic loci, to determine what concomitant mutations are associated with IFNα treatment outcomes. This is important as, in the long-term, concomitant mutations in genes, including *TP53*, *EZH2* and *ASXL1*, confer a higher risk of disease transformation to phenotypes with poor clinical outcomes, such as MF and AML^1^. Consequently, these studies are vital for determining the potential of IFN therapy to delay or prevent disease progression.

## Conclusions

In summary, the clinical management of patients with MPN has been dominated by three main approaches: chemotherapy for cytoreduction, targeted JAK2 inhibition or long-acting IFNα analogues. Although all are highly effective, to date none have shown the sustained ability to modify the natural history of disease and prevent transformation to sAML or sMF. Long-acting pegylated IFNα is the only therapy to show reliable and deep molecular responses, but requires long-term treatment and is often limited by toxicity. Studies, like Saleiro et al., have identified promising candidates that may synergise with IFNα by targeting stem cell function or feedback loops that mediate treatment resistance. The next phase of clinical studies should address rational combinations and the contribution of concomitant mutations to treatment response, with ambitious clinical endpoints, including molecular remission, treatment free remission and prevention of disease transformation.
